# Early-Life Adversity Predicting The Incidence of Multisite Chronic Pain in The General Population

**DOI:** 10.1192/j.eurpsy.2026.11482

**Published:** 2026-07-31

**Authors:** I. Rouch, M. P. Strippoli, B. Laurent, J.-M. Dorey, M. Preisig, A. von Gunten, M. Preisig

**Affiliations:** 1 Saint-Etienne university hospital, Saint Etienne, France; 2 CHUV, Lausanne, Switzerland; 3 U, INSERM; 4 CH Le Vinatier, Lyon, France

## Abstract

**Introduction:**

Adverse childhood events (ACEs) have been linked to widespread chronic pain (CP) in various cross-sectional studies, mainly in clinical populations. However, the independent role of different ACEs on the development of different types of CP remains elusive. Accordingly, we aimed to prospectively assess the associations between specific types of ACEs with the development of multisite CP in a large population-based cohort.

**Objectives:**

Accordingly, we aimed to prospectively assess the associations between specific types of ACEs with the development of multisite CP in a large population-based cohort.

**Methods:**

Data stemmed from the three first follow-up evaluations of CoLaus|PsyCoLaus, a prospective population-based cohort study of initially 6734 participants (age range: 35-75 years). The present sample included 1537 participants with 2161 analysable intervals (49.7% men, mean age 57.3 years). Diagnostic criteria for ACEs were elicited using semi-structured interviews, CP was assessed by self-rating questionnaires. Multinomial logistic regressions with Generalized Estimating Equations method analysed the relationship between the different ACEs measured in the beginning of the interval and the risk of developing multisite CP during the follow-up. Sensitivity analyses were performed to assess the predictive value of ACEs on multisite CP with neuropathic features.

**Results:**

Participants with a history of parental divorce or separation had an increased risk of developing multisite CP at during follow-up in comparison to those without (RR1.98; 95% CI 1.13-3.47). A strong association was highlighted between parental divorce or separation and the risk of subsequent CP with neuropathic characteristics (RR 4.21, 95% CI 1.45-12.18).

**Conclusions:**

These results highlight the importance of psychotherapeutic management of people experiencing parental separation to prevent CP, in the future.

**Disclosure of Interest:**

None Declared

